# What Can Be Learned from the Early Stages of the COVID-19 Vaccination Rollout in Australia: A Case Study

**DOI:** 10.3390/epidemiologia2040040

**Published:** 2021-12-03

**Authors:** Juliette Caroline Choiseul, Paris Jade Emmerson, Turan Eslanloo Pereira, Seyed-Moeen Hosseinalipour, Jennifer Hasselgård-Rowe

**Affiliations:** 1 Institute of Global Health, University of Geneva, 1202 Geneva, Switzerland; juliette.choiseul@etu.unige.ch (J.C.C.); paris.emmerson@etu.unige.ch (P.J.E.); turan.eslanloo@etu.unige.ch (T.E.P.); seyed-moeen.hosseinalipour@etu.unige.ch (S.-M.H.); 2 Global Studies Institute, University of Geneva, 1205 Geneva, Switzerland

**Keywords:** vaccine strategy, AstraZeneca, COVID-19 response, Australia, federal/state government, economy, supply strategy, Aboriginal populations

## Abstract

This paper addresses the SARS-CoV-2 vaccination progress in Australia. Globally, Australia was initially praised for its national COVID-19 response, reflecting well with regard to case numbers and mortality rates. However, Australia’s progress with its vaccine rollout has come under scrutiny. When compared globally, it fares very low in terms of the number of vaccine doses administered. This paper discusses the first three months of the vaccination process, and the challenges Australia faced during that time. Through an extensive literature review, data was collected on relevant topics concerning all aspects of the Australian COVID-19 situation. The following key points are discussed: the specific COVID-19 organisation at the federal vs. the state government levels, the Australian economy, the vaccine supply strategy, and the vaccine priority roll out. In conclusion, we highlight the impact of Australia initially relying heavily on the AstraZeneca vaccine, which subsequently came under fire regarding safety issues likely linking the vaccine to thrombosis with thrombocytopenia syndrome (TTS).

## 1. Introduction

The COVID-19 pandemic, as a public and global health emergency, has provided experts with a unique opportunity to apply a critical eye to pandemic preparedness plans (and subsequently, vaccination strategies) of governments around the world. As an isolated island nation with a strong economy and well-structured government pandemic plan, Australia was uniquely prepared to respond to the singular COVID-19 pandemic which began in early 2020. Australia received special recognition for its seemingly smooth handling of the pandemic before the development and distribution of vaccines [1]. Part of this could be attributed to the zero-tolerance policy which was adopted by the government shortly after COVID-19 cases began to spike in early 2020. This zero-tolerance policy involved shutting national borders to anyone except returning Australians, and is still in place with certain exceptions, even if voices are now calling for a change of strategy [2]. This policy also involved putting people in mandatory hotel quarantines for 2 weeks, in some cases at least partially at returning citizens’ own expense [3,4]. This policy, combined with the swift implementation of rolling out lockdowns wherever and whenever necessary, has enabled Australia to keep the sheer number of positive COVID-19 cases down, compared with other high-income countries. The emergence of the Delta variant has significantly changed the epidemic situation in Australia with the number of daily cases currently rising (at the time of writing, in early September 2021). There were a recorded 1356 cases on the 30th of August 2021 and the total number of deaths stood at 1006. Before this third wave, the numbers remained consistently low. Even the second wave between June to October 2020 did not compare with this current number of infections, with the peak number of daily cases reaching 721 on the 30th of July 2020 [5].

Additionally, the Australian economic response has protected its economy in ways that have been unmatched by many other countries, and have allowed for recovery in early 2021 [6]. However, it is clear that the Australian vaccination scheme has not achieved the same success as the Australian economy. With a vaccination rate on the 30th of August 2021 of 74 doses per 100 people and only 27.3% of the population fully vaccinated, these numbers fall short of Australia’s originally stated goals and puts it behind its peers [7]. For all intents and purposes, the inception of the Australian vaccine rollout encountered many obstacles and could be deemed a failure. The heavy reliance on AstraZeneca backfired, as rising concerns regarding the safety of the vaccine have forced the government to revise its entire vaccination strategy. In order to fully analyse the situation at the early stage of the vaccination rollout, and provide insight into future movements by concerned parties, this paper aims to critically analyse the administrative choices made throughout 2020 and leading up to the end of May 2021 regarding key areas of the Australian coronavirus response. These areas include the Australian federal and state distribution of administrative powers, the effect and mitigation of the pandemic on the Australian economy, and the stated national vaccination strategy. Throughout, this paper will also place special emphasis on the needs and prioritization of Aboriginal Australians, who, historically, constitute a more vulnerable population.

## 2. Materials and Methods

For this paper, a narrative literature review was performed on a wide variety of data sources published until the end of May 2021. These sources included government reports, legal briefs, published papers, and Australian public news outlets. Particular focus was given to the following areas of interest: Australian public health regulations, demographic and epidemiological data, economic statistics, and data concerning Australia’s pharmaceutical industries.

Government reports and governmental Department of Health websites constituted the primary sources of data with regard to the vaccination rollout and priority groups, as well as the political organization of the vaccination programmes [8]. Data on the Australian demography as well as epidemiological statistics related to the COVID-19 outbreak in 2020 were retrieved from the Australian Bureau of Statistics online resources [9]. News media releases were used to follow the evolution of the vaccination rollout and information surrounding vaccine manufacturing and sourcing. The main newspapers regularly consulted were The Sydney Morning Herald, The Guardian, and ABC Australia due to their extensive coverage of the COVID-19 pandemic and of the vaccination programme. A lot of the information about the economic impact of the pandemic and its consequences was retrieved from the financial newspaper Reuters, widely known for its impartiality.

Secondary sources of data were found through search engines such as PubMed and Google Scholar. These data were used to create a comprehensive case profile of the Australian situation at the beginning of the COVID-19 pandemic, which was then used to review and analyse the Australian COVID-19 response and vaccination strategy.

## 3. Results

### 3.1. Case Presentation

At the end of 2020 or at the latest since early 2021, most high-income countries started rolling out mass vaccination campaigns against COVID-19. With the progress of vaccine manufacturing, countries enhanced their vaccination capacities and rapidly immunised their most vulnerable and exposed populations, and quickly moved to vaccinating the more general populations. At the end of May 2021, 72.3% of the adult population in the UK had received at least one dose of the vaccine. This number was 50% in the USA, and most Western European countries had administered around 50 doses per 100 inhabitants, and were observing a regression of the epidemic with a decrease in the incidence and hospitalizations and mortality rates [10]. In contrast, Australia had administered 14 doses per 100 inhabitants, and only 1.6% of its population were fully vaccinated [10]. To better understand the reasons and potential impact of this delayed rollout, but also the political choices around the vaccination strategy, in particular regarding the definition of priority groups, some of the specificities of the Australian situation need to be discussed first. Australia’s geographical situation, demography, and political organization as a federal state greatly influenced the design of the vaccination program. On the other hand, Australia’s response to the COVID-19 outbreak and the resulting epidemiological and economic situation are key to understanding the impact that the delayed rollout might have on the Australian economy and daily life. Therefore, these aspects are covered in this first part of the present paper.

Australia is one of the biggest countries in the world with a total area of 7,692,000 km^2^ for 25 million inhabitants, 3.3% of whom are of Indigenous origin [11,12]. The term ‘Indigenous Australians’ is used to refer to persons who are of Aboriginal or Torres Strait Islander origin. Australia has a constrictive pyramid of ages with a total fertility rate of 1.8 live births per woman and a median age of 37.8 years in 2016, which is 6 years younger than the Western European population [12]. It is interesting to note that the Indigenous population has a completely different demography to the non-Indigenous population, with a median age of 26.0 years and 2.3 live births per woman [13]. The proportion of Indigenous people of 65 years of age or more is 4% whereas it is 16% for the total population. In the context of COVID-19, this feature should be advantageous to Indigenous populations. However, Australian Indigenous populations are more likely to suffer from underlying conditions that increase vulnerability to disease severity. Indigenous Australians mostly reside in urban and regional areas but a considerable proportion still live in remote areas [13].

Politically, Australia is run as a federal parliamentary constitutional monarchy composed of six states, and two internal territories. Under the federalist system, each jurisdiction has their own parliament and constitutions and can create state specific laws within their own geographical territory. The two mainland territories (the Northern Territory (NT) and the Australian Capital Territory (ACT)) have been given the right to a limited amount of self-governance from the federal government, controlled by a locally elected government [14]. The federal government governs the overall economy, trade, immigration, foreign affairs, and social security of the country, whereas State and Territory governments have a more focused responsibility with regard to their populations, such as for public health, town planning, emergency and community services. In terms of vaccination governance in general, the National Immunisation Program (NIP) provides comprehensive guidelines for the Australian population of when to have their vaccines [15]. All vaccines are free, and target vulnerable populations including children up to 16 years, the elderly, and pregnant women. In addition, there are three schedules in place that aim for inclusiveness: for the “non-Indigenous” population [16], for “all Aboriginal and Torres Strait Islander people” [17] and one for “all people” [18]. Overall, Australia’s immunisation rates against vaccine preventable diseases (including the Indigenous community) are very high [19], contributing to the unlikely risk of an outbreak of infectious diseases within the country [20]. This was partly achieved through the no jab, no pay policy, introduced in 2015, that denies family assistance payments to families whose children are not vaccinated according to the National Immunization Program. The States of New South Wales, Queensland, Victoria, and Western Australia, also implemented a no jab, no play policy, which means that children who are not immunized are not allowed to attend preschool and childcare centres [21].

In November 2020, the Australian federal government published Australia’s COVID-19 Vaccination Policy [22]. It includes figures for Australia’s entire population, within each phase. Each State and Territory is then responsible for modifying its own internal programs which must ultimately be aligned with the national vaccination policy. According to the Australian COVID-19 Vaccination Policy, the federal government is accountable for the following issues: setting funding policies, issuing national communications, regulating vaccine safety, overlooking the manufacturing process, storage, intrastate and interstate vaccine transportation, and supervising data including vaccination rates [22].

Australia’s first COVID-19 case was detected in January 2020 in Victoria in a traveller coming from Wuhan, China. Up until May 2021, the country experienced two waves of infections. One in March 2020, when the daily number of new detected cases peaked at 458 in one day, and a second one during the austral winter in July 2020, when up to 721 cases were reported in one day. In the early days of the vaccination campaign, in May 2021, a total of 30,004 cases were detected since the beginning of the pandemic and there were 910 deaths to mourn in Australia. At the same date, the 7-day average of new detected cases was 82, with the majority being acquired overseas and contained in quarantine hotels hosting citizens and residents coming back from abroad [23]. According to the National Indigenous Television, the infection rates were extraordinarily low among Indigenous populations, with no reported mortality or hospitalizations [24]. This is an important achievement considering previous experiences such as the influenza H1N1 2009 pandemic in North Queensland, where there was a huge gap between Indigenous and non-Indigenous populations [25]. The current reverse gap in COVID-19 cases between Indigenous and non-Indigenous populations in Australia is believed to be mostly the result of Indigenous-led responses to COVID-19 in these communities [26].

### 3.2. Management and Outcome of the COVID-19 Outbreak

From the outset, the federal government adopted a strict zero tolerance approach, and closed its national borders on the 20th of March 2020. Australia’s international borders have remained closed with strict entry restrictions since, in fact constituting measures that go against the World Health Organisation’s (WHO’s) recommendations [27]. Citizens and permanent residents are not allowed to leave the country without an exemption to travel except for some selected destinations [1]. The number of Australian citizens and residents wanting to return to the country has been restricted. In April 2021, this figure stood at 40,000 people who were still registered to receive assistance from the Department of Foreign Affairs and Trade (DFAT) to return to their country [28]. Very strict quarantine measures including a 14-day hotel quarantine at individuals’ own expense are applied to people entering the country.

Internally, each State and Territory has the right by law to close its own borders, and apply entry requirements as it wishes. The situations regarding state borders have been known to change at very short notice, and are ultimately responsive to frequently changing circumstances [29]. Other non-pharmaceutical measures such as confinement, where people were not allowed to leave a certain perimeter, except for essential activities, have been applied and an efficient system of contacts tracing was put in place. Massive testing and targeted lockdowns were also part of Australia’s response to the pandemic [1]. Epidemiology at a state level is key to provide information to evaluate state coordination and response mechanisms. As seen in Table 1, the highest number of cases have mostly been concentrated in the Eastern part of Australia: in Victoria (20,547 cases) and New South Wales (5570 cases).

#### 3.2.1. Organization between the Federal and State Governments

Since COVID-19 constitutes a public health crisis, official responsibility lies with the States and Territories to deal with the situation [30]. However, COVID-19 is not solely focused on negative health outcomes, but also impacts on Australia’s economy. Therefore, this creates a certain obligation for the federal government to take action.

On the 13th of March 2020, the National Cabinet was created by the Australian Prime Minister, as part of the government’s response to the pandemic, to ensure effective communication and coordination between the Prime Minister himself, the State premiers, and the first ministers of Territories. It allows for horizontal communication across all the jurisdictions on COVID-19-related issues. Unlike in Parliament, where States are represented relative to population size, the national cabinet provides for equal representation and communication across States and Territories [31]. The National Cabinet has been seen as a reform of the historical Council of Australian Governments (COAG), which was more of an overarching communication system, and has been recognised as the peak body of Australian federalism [32]. The Organisation for Economic Co-operation and Development (OECD) praised this decision by Australia, and highlighted the importance of ad hoc structures, and crisis decision-making being completed at a high level, and then supported by existing structures [33].

#### 3.2.2. Economic Burden

##### Before COVID-19

Australia has the 12th largest economy in the world [34], and until 2020, the country had not experienced a recession since 1990, which is the longest interval recorded for a developed country since the second World War [35]. The national growth in GDP, which had previously been steadily increasing, began to level off in 2018 and 2019, but the economy was predicted to grow at a moderately fast rate in 2020, with the IMF predicting a GDP growth rate of 2.0% [36]. Australia exports many goods and services but as an island nation, Australia relies heavily on imports, and the margin between its imports and exports only makes up 0.5% of the GDP in favour of exports [37]. Notable for the purpose of this paper, one of the larger classes of goods imported into Australia are medical equipment and some pharmaceuticals [38]. Australia’s in-house pharmaceutical industry is still developing, which can be seen from the fact that in 2017, Australia, most of Asia, and Africa combined made up only 17% of global pharmaceutical sales [39]. The Australian pharmaceutical industry will be explored in depth in the next section of this paper. Additionally, the role of tourism in Australia’s economy is notable. Whereas tourism made up a relatively modest 3.1% of the country’s economy in 2018–2019 [40], a large part of this tourism was academic tourism, with students coming from around the world to study at Australian universities. This activity made up 40% of the country’s service exports [41].

##### 2020. Economic State

Whereas the Australian economy was poised to make a modest come-back from a stagnating GDP in the first quarter of 2020, the arrival of the SARS-CoV-2 virus to the country’s shores on the 25th of January 2020 [42] constituted an obvious damper on economic activities. By mid-March, non-essential businesses had already been closed, and these changes showed in the country’s slightly decreasing GDP [43]. Soon after, the country closed its borders entirely, effectively shutting out international tourism [44]. The Australian government acted quickly in light of these events and drafted and confirmed a stimulus package of AUD 17.6 billion, which was focused on preventing job losses and helping medium-sized to small businesses stay afloat [45]. These actions set a theme in the Australian government’s role in maintaining the economy, as throughout the pandemic various financial assistance programs would be reinforced, made more accessible, or expanded. In addition, the government announced a new wage subsidy program called Jobkeeper, which would pay employers 70% of national median wages for each qualifying employee every 2 weeks [46]. The Jobkeeper program began in March 2020, continued throughout the year, with gradual cut-backs as the economy recovered, and by April 2021 was officially discontinued [47]. Complementary to Jobkeeper was Jobseeker, a continuation of existing social security measures for unemployed/underemployed adults. This service was also expanded during the pandemic, and qualifying Australian residents received an extra stipend bimonthly, starting at AUD 550 and decreasing to AUD 250 later on in the year [48,49]. Whereas the number of new COVID-19 cases was decreasing by the end of April, and would not spike again until July 2020, the Australian economy struggled throughout the first half of 2020, due to losses in revenue and slowly increasing unemployment. In June, the national GDP fell by 7%, and national unemployment reached a peak of 7.5% in July [43]. Meanwhile, the new COVID-19 cases were increasing daily again, reaching their all-time high at the end of July. By August, job vacancies began to recover, but the overall economy was still in difficulty. In coordination with a rapidly decreasing number of new daily cases in that month [50], September saw a 3.4% rise in GDP [43], and the second wave of COVID-19 was over by the beginning of November 2020.

Since the end of the Australian second wave in September 2020, the country has seen low daily numbers of new cases, which have been met with swift and strong responses from the State governments, with many regions having “snap” lockdowns, which arrived swiftly and with little notice, but only lasted a few days [51]. As a result, both local and national economies have been able to benefit from a stable environment for making a modest recovery. December 2020 saw a 3.1% increase in the national GDP, despite the fact that the country’s continued border closure had left some industries, such as tourism and hospitality, with lower levels of employment. In January 2021, 93% of the jobs that had been lost due to COVID-19 had been recovered [43].

With regard to vaccine production, the Australian government devoted AUD 1.9 billion to ensure access to vaccines, with a large part of this budget going towards agreements for the domestic production of the AstraZeneca vaccine [52]. Whereas the pay-off of this investment is yet to be determined, it seemed clear during the first semester of 2021 that the Australian economy was already back on track towards normality, even without a successful mass vaccination program. Even the tourism industry was likely to show marginal improvement, as Australia opened travel to and from its close ally, New Zealand, in April 2021 [53]. However, Australia will not be able to return to a growth position without opening its national borders fully, a situation which is unlikely to happen before the end of 2021 [54]. Another consideration is the position of the government to release aid as rapidly and reliably as it did. Whereas the Australian stimulus and aid packages played a huge part in keeping the economy stable, the Australian government had much lower national debt than other developed countries, and thus may have been in a uniquely good position to do so [55]. Seeing as the government will likely need to invest even more money into in-house vaccine production now that there is a need to shift to mRNA vaccine production [56], this will likely add to the government’s debt, causing anxiety for the state of the economy. Whereas the government’s stance on this spending is that extraordinary allowances should be made for this “once-in-a-century shock” [57], it is becoming increasingly obvious that the coronavirus situation in Australia will have lasting effects for several fiscal years, and the only holistic and viable solution is to rectify the vaccine rollout as soon as possible to try to achieve pre-COVID normality again.

### 3.3. Vaccination Strategy

As detailed in Table 2, the Australian vaccination rollout plan consists of a 5-step plan in the prioritization of the populations who should receive the vaccine first.

Given the controlled epidemiological situation in Australia in early 2021, and the fact that most of the cases were imported, in Phase 1a, the vaccination targeted workers with high exposure risk to potential cases coming from abroad, namely border workers and employees working in quarantine hotels. This is quite specific to Australia, to its epidemiological situation, and the way risks of outbreaks have been handled according to the zero-tolerance policy. Phase 1a also included frontline health care workers, and people in care residences where many outbreaks have occurred worldwide. During Phase 1a, 678,000 persons were expected to be vaccinated with two doses, unless they had already been infected in the past, then they were to receive one dose. Phase 1b focused on the people most at risk of developing a severe form of the disease, namely elderly people or adults with underlying medical conditions, but also Aboriginal and Torres Strait Islander people aged 55 or above. This priority in vaccination for Indigenous people is due to their higher risk of suffering from underlying conditions and having less access to health care, higher risk of chronic diseases than their non-Indigenous compatriots [59], greater risk of death and hospitalization [60], increased chances of living in inadequate housing conditions [61], and higher risk of hazards coming from food insecurity and lack of access to clean water and sanitation [61]. This is in line with the Indigenous Australians’ Health Programme (IAHP) whose objective is to ensure access to adapted health care services [62]. Phase 1b also covered all the remaining critical and high-risk workers such as people working in emergency services, the remaining health care workers, and those working in the meat processing industry. A total of 6,139,000 people should have been immunized in this phase [63]. The 6,570,000 candidates for Phase 2a vaccination were all the remaining critical and high risk workers, such as supermarket workers and people working in schools and child care [63], people aged 50 and above, and all the adult Aboriginal and Torres Strait Islanders. The rest of the adult population (6,643,000 persons) was also meant to be immunized in Phase 2b. The rollout program also tentatively included children in Phase 3, although the clinical trials of the vaccine candidates had not yet been completed on this population when the vaccination strategy was made public.

In most high-income countries, the vaccination campaign against COVID-19 started in late 2020 or early in 2021. Australia kicked off its vaccination campaign on the 22nd of February 2021, after the provisional approval of the Pfizer/BioNTech vaccine on the 25th of January 2021 [64] and of the AstraZeneca vaccine on the 16th of February 2021 [65]. The initial program was to deliver 4 million doses of vaccines (Phase 1 and beginning of 1b) before the end of March 2021, relying on 3.8 million doses of the Oxford/AstraZeneca vaccine manufactured in the European Union and 700,000 doses of Pfizer/BioNTech being imported from the UK. The complete rollout amongst the adult population was meant to be completed by October 2021, mainly with the Oxford/AstraZeneca vaccine manufactured in Australia [66]. Unfortunately, these time targets rapidly became unrealistic, as will be discussed in the following sections.

#### 3.3.1. Regulations of Vaccines

In Australia, a key federal responsibility is to ensure population safety. Specifically looking at COVID-19 vaccinations, Australia’s Department of Health (DOH) provides many branches of organisations, and employs a lot of individuals who guarantee safety through maintaining vaccine regulations. The Australia Technical Advisory Group on Immunisation (ATAGI) supplies advice surrounding COVID-19 immunisation programs and policies to the Australian Government Department of Health and the Australian Minister of Health.

The Therapeutic Goods Administration (TGA), part of the Australian DOH, works as a key vaccine regulator to guarantee the efficacy, quality and safety of vaccines before they are allowed to be used within Australia [67]. During the production of COVID-19 vaccinations, the TGA ensures quality standards through assessing every vaccine batch before they are able to be used in Australia [68]. The TGA works closely with the European Medicines Agency’s international guidelines to weigh associated risks compared with the benefits for each vaccine [68]. At a national level, a provisional approval pathway is in place, where if the vaccine benefits outweigh the risks, then the vaccine will be registered on the Australian Register of Therapeutic Goods, and ready for use [67]. By May 2021, only two vaccines had been approved for use in Australia by the TGA (Table 3).

Since the approval of the COVID-19 vaccines in Australia, safety monitoring through the ATAGI and the TGA has been a continuous effort, as a responsibility of the federal government. After the global panic surrounding the safety of AstraZeneca [70], the nation’s top vaccine and medical regulators met to discuss the risks associated with the AstraZeneca vaccine. On the 8th of April 2021, changes were made to the vaccination rollout guidelines and communicated to the Australian public, advising that only people over 50 years old would be receiving the AstraZeneca vaccination, due to safety concerns. The Australian Prime Minister addressed the public in a press conference emphasizing that public trust is key for the national vaccine programme to work. A total of six cases of thrombosis with thrombocytopenia syndrome (TTS) were reported in Australia following the administration of the AstraZeneca vaccination, with five reported in persons 50 years old or younger [71].

Some countries such as the UK, France, and the USA have been implementing vaccine injury compensation programs (VICPs), even before the COVID-19 pandemic broke out. Such programs mean that if an individual suffers from adverse side-effects following a vaccination, he or she can ask for compensation without having to go to court or provide evidence of negligence or any other kind of fault. According to this model, countries assume the liability in case of adverse side-effects from the vaccination and provide funding to compensate the person in question. Some countries have implemented VICPs following the COVID-19 pandemic to accelerate their vaccine rollouts, and the COVAX facility [72] has created a global mechanism to allow 92 low- and middle-income countries to benefit from such programs [73].

The implementation of such a non-fault compensation program provides a guarantee to individuals that they will be covered in case of a health problem following the vaccination. This can be seen as a possible tool to incentivize the population to become vaccinated and limit vaccine hesitancy [74]. It also has the effect that the vaccine manufacturers are less at risk of being sued after an adverse event and might feel more comfortable to deploy their vaccines in countries that have put in place these compensation schemes.

Australia does not have such a program in place [75]. This may hinder Australia from being prioritized over other countries with regard to pharmaceutical companies’ vaccination supply strategies [74,76]. It may also restrict access to some vaccines. For instance, no agreement was concluded with regard to the Johnson and Johnson vaccine, even before the concerns about possible links between this vaccine and TTS arose in part because the company required a non-fault compensation scheme that Australia was not prepared to provide [77].

#### 3.3.2. Storage, Transportation and Dose Allocation of Vaccines

With regard to the storage and transportation of vaccines, Australia’s National Cabinet has aimed to create more of a coordinated response through the federal government, States, and Territories working interactively. For example, a State organizes the vaccination site, which then has to be agreed to by the Australian Government, so as to align it with the jurisdictional implementation plan. This then allows the federal government to be in charge of the storage and transportation of the vaccines, ensuring accurate distribution to the correct facilities within the States and Territories. Once the vaccine is delivered to the site, then responsibility for the storage, handling, and overall physical safety of the vaccine changes to the State. The management and arrangements of each vaccination site (including providing the appropriate training, allocating staff, and ensuring the correct data is communicated to the federal government) then becomes the sole responsibility of the State [22].

The federal government entrusted the logistics companies DHL and Linfox with the responsibility for the storage and distribution of all the vaccines throughout the territory, as well as for the related medical equipment such as syringes, needles, and personal protection equipment [78]. The task is an unprecedented challenge for the Pfizer/BioNTech mRNA vaccine which requires a strict control of the cold chain, especially given Australia’s geography and the necessity to reach very remote areas. The Australian agreement with Pfizer includes the supply of purpose-made cold containers for the adequate transportation of the vaccine doses, but the monitoring of the vaccine storage temperature needs to be ensured by the transporters.

The dispatch of the mRNA vaccine to the most remote areas in Australia is being facilitated by the installation of adapted freezers in a central hub from which the vaccines can then be transported to immunize the populations of the most remote areas. In the Torres Strait Islands for instance, the freezer is installed on Thursday Island and is used to store the doses necessary for the neighbouring islands [79]. In early April 2021, following new evidence, the TGA approved more flexible storage conditions for the Pfizer/BioNTech vaccine, allowing unopened vials to be kept at −25 °C to −15 °C for as long as two weeks, which is a temperature range attainable by a domestic freezer. Doses can even be stored at positive temperatures, up to 8 °C for a maximum of 5 days [80]. This simplifies the logistics around vaccine storage and distribution to remote areas considerably.

The tracking of doses and the follow up of the vaccination rollout data was entrusted to the company Accenture, which developed a solution especially designed for the distribution of COVID-19 vaccines. The software developed by the company also keeps track of any reported side-effects.

With limited doses of vaccines available, resource allocation is crucial within Australia as the vaccines must go to the most vulnerable first. In accordance with the COVID-19 policy, the allocation of vaccine doses for each jurisdiction is subject to the number of residents within the relevant phase of the priority roll out [22]. The policy also takes into account the varying incidences of COVID-19 across jurisdictions, with additional doses provided to a certain State or Territory if and where needed, if national stock levels are able to do so [22]. Throughout its COVID-19 management, communication from the States and Territories has been made paramount by the Australian government, and includes accurately reporting numbers of doses administered, the stock levels and also any wastage that has occurred [22]. Ultimately, a Chief Medical Officer in each jurisdiction is responsible for disseminating information to the National Cabinet.

Table 4 shows the distribution of vaccines to each jurisdiction during the early stage of the vaccination rollout in May 2021. Interestingly, Victoria has received the highest number of vaccines, even though it has the second largest population. This is no doubt a reflection of past issues, as the State had the highest number of COVID-19 cases and deaths, and 75.4% of cases stemmed from locally acquired infections [81].

The vaccination rollout strategy in Australia is based on a personalized approach by general practitioners (GPs) within the communities [83]. Vaccination sites vary between jurisdictions; however, they have to be in line with the requirements of the Australian government [84]. For example, within NSW, the vaccination site locations were kept relatively private, offering a small number of locations within the state. The state government collaborated with companies to target and send invitations for vaccination to eligible staff members within priority groups in the Phase 1a/1b rollout [85]. The NSW government states that with improved access to resources, more locations will become available, aiming for it to be after phase 1B onwards [86]. States have also implemented vaccination hubs to accelerate the vaccination rate, but the majority of doses are expected to be administered by the GPs. This strategy presents the advantage of allowing a face-to-face communication with a potential vaccination candidate to improve confidence and convince a larger part of the population to become vaccinated. The downside is probably a more complex logistics chain and a potential bottleneck at the moment when vaccine manufacturing and supply have been successfully scaled up. This, however, was not the case at the early stages of vaccination in May 2021 [87]. In addition, depending on the vaccines used, maintaining the cold chain can be a challenge in non-specialized centres.

#### 3.3.3. Australian Pharmaceutical Industry

Australia has a long history of successes in vaccination campaigns and vaccination manufacturing. Already in the middle of the 19th century, Australia started producing a vaccine against smallpox, being one of the first countries in the world to kick off the manufacturing of vaccines [88]. Since then, Australia has continued producing most of its vaccines in-house. According to a recent report from the Institute for Integrated Research [89], Australia imports more than 90% of the medicines used in the country and has significantly reduced its manufacturing capacity for active ingredients, including most of the medicines on the WHO list of Essential Medicines. The country relies on complex supply chains for the delivery of medicines, at times experiencing potentially critical shortages. Various pharmaceutical companies such as GSK and Pfizer have very recently announced the closure of production sites in Melbourne and Perth, reducing Australia’s medicine manufacturing force even further. The notable exception to this trend is the production of vaccines. Throughout the years, Australia has maintained its industrial manufacturing capacities for vaccines with the main actors in this field being GSK and CSL, one of the leading companies in influenza vaccine production.

#### 3.3.4. Vaccine Sourcing and Domestic Production

Since the pandemic began, Australia has been very active in the vaccine race and entered into several agreements with manufacturers and research groups in order to ensure it would be able to immunize its population against COVID-19. As a result of all of the different agreements, Australia is one of the countries with the highest number of doses (five) secured per inhabitant [90,91].

Australia’s vaccine sourcing strategy consisted of investing in vaccines coming from various technologies in order to diversify the portfolio and increase the chances of success. The country however did not enter into agreements for every potentially successful vaccine candidate and initially picked only one vaccine of each type; the Pfizer/BioNTech vaccine for mRNA technology, the University of Oxford/AstraZeneca one for the adenovirus vector technology, and the Novavax Inc, a protein vaccine [83,92]. Australia also joined the COVAX facility, the global initiative from WHO, Gavi, and the Coalition for Epidemic Preparedness Innovations aiming to make vaccine access equitable around the globe, with an upfront payment of AUD 123.2 million and AUD 80 million invested in the Advanced Market commitment of COVAX facility [92]. In total, Australia has so far invested AUD 3.3 billion in purchasing vaccines [92]. On top of that, the country has spent AUD 363 million to support research and development [92].

Australia also tried to support an internally developed vaccine from the University of Queensland and had even entered into an agreement for a purchase of 51 million doses, to be produced by CSL, back in September 2020 [93]. The first doses were expected to be available by mid-2021. However, this vaccine candidate was not successful, and was abandoned following Phase 1 clinical trials.

At the time of the beginning of the national vaccination campaign in February 2021, Australia had secured 20 million doses from Pfizer/BioNTech, 50 million doses of the Novavax vaccine, 25 million doses from the COVAX facility, and 53.8 million doses from Oxford University/AstraZeneca vaccines, of which 50 million were to be manufactured in Australia by CSL [92]. However, except for 3.8 million doses of the Oxford/AstraZeneca vaccine and 10 million doses of the Pfizer/BioNTech vaccine, the vaccines purchased by Australia were not expected to be available before mid-2021 and the vaccination rollout strategy was relying heavily on the onshore manufactured vaccines. The rollout plan was to start the vaccination campaign in early 2021 with imported vaccines, whereas the CSL plant was ramping up the domestic production. Thanks to these imports, the health authorities were expecting to have 4 million doses injected by the end of March 2021. In the end, only 870,000 doses of Pfizer/BioNTech and 700,000 doses of AstraZeneca were delivered on time, significantly delaying the vaccination campaign [94].

Producing the vaccine onshore appears to be important to the Australian government, in order to reduce the dependency of the country on imports from foreign countries. This may explain the decision to back the Queensland University vaccine candidate and the AstraZeneca production by CSL in Melbourne. This issue has grown in importance since the European Commission blocked an export of 250,000 doses of the vaccine manufactured by AstraZeneca in its European production plants resulting in a lack of confidence from the Australian government concerning future deliveries [83,92,95].

The domestic production of the 50 million doses in 2021 of the Oxford University/AstraZeneca vaccine was meant to be the angular stone of the Australian vaccine supply strategy. However, this plan fell through when the ATAGI advised that the Oxford/AstraZeneca vaccine should not be used for people under 50 years of age, unless the benefit of the vaccination clearly outweighs the associated risk [96]. Consequently, the initial target of having the whole population vaccinated by the end of October 2021, as well as the rollout plan had to be reviewed in the absence of a plan B.

The first move from the commonwealth government, after the ATAGI announcement, was to purchase an additional 10 million doses of the Pfizer/BioNTech vaccine, but they are not expected to be delivered before the end of 2021 [97]. In parallel, the federal government decided to invest in the building capability of mRNA vaccine production, and the State government of Victoria, released a funding of AUD 50 million to develop internal large-scale manufacturing for mRNA vaccines [98]. These measures secured enough doses to vaccinate the whole adult population in Australia, even if they cannot enable the country to meet its initial vaccination time targets. They will also strengthen Australia’s position for future vaccination campaigns and vaccine development.

## 4. Discussion

Compared with the praise Australia initially received for its COVID-19 management, the country’s position with regard to its vaccination rollout strategy has fallen far below target. The pandemic response resulted in the creation of a National Cabinet which can be seen as a success. However, organisational issues within the various Australian governments are not unfamiliar. Interestingly, vertical and shared coordination and communication strategies—a main objective of the Cabinet—are questionable with regard to the vaccination rollout.

The COVID-19 response at the State and Territory levels, can be seen as effective. Fast lockdowns, mandatory quarantines and track and trace systems managed to keep infections low and controlled for several months. However, at the beginning of the vaccination rollout, State governments were heavily criticized for their management, especially by the community saying that the rollout was too slow, with certain high-risk individuals who were still not vaccinated by the end of May 2021. As the rollout was still in Phase 1a and 1b for all States, this can therefore be seen as a problem coming from the overarching government. Furthermore, the governments of NSW and Queensland have publicly criticised the federal government, stating it is to be blamed for the slow progress within their communities. The Australian government also promised domestic production and that in-house production would meet the target of one million doses per week. As manufacturing started in March 2021, this has also been another target which has not been met by the federal government. Moreover, the fact that the federal government has held off sharing accurate information on the number of vaccines available has only created mismatched organisation and public dissatisfaction with regard to the vaccination campaign.

Whereas the Australian economy’s success has been well documented, there are some weak points which have been highlighted throughout 2020. For example, the Australian COVID-19 situation exposes a logistic/supply chain issue inherent to the Australian pandemic response due to the country’s isolation. It also reveals a huge vulnerability inherent to Australia’s profile, namely that it is, functionally, at the end of the supply chain for many essential goods [99]. This is illustrated by the fact that, on the 4th of March 2021, Italy blocked a shipment of 250,000 doses of the AstraZeneca vaccine, as the European bloc reserves the right to do so under scarcity conditions [100]. Exacerbating this supply chain issue is the country’s relatively underdeveloped pharmaceutical industry, which often does not have the capacity to make up for the gap even in regular conditions, as shortages of other medications such as EpiPens have occurred in recent years [101].

In this regard, the strategy of the Australian government to push for onshore production of vaccines seems reasonable, given the global pandemic situation and the race most wealthy countries entered in to secure enough vaccines to immunize their populations. The delay in vaccine delivery, due to the supply chain difficulties described above, confirms the appropriateness of this approach. However, given the Australian history of innovation and large-scale manufacturing in vaccines, the question of why Australia has invested significantly less in the COVID-19 vaccine than other high-income countries may be raised.

It is very easy to make recommendations or criticize a country’s actions in hindsight, especially regarding an unprecedented situation such as the COVID-19 pandemic. However, given the very aggressive and initially successful response of Australia to the COVID-19 epidemic, the government’s investment in the vaccination campaign is relatively low. Especially when compared, for instance, with the USD 18 billion invested by the USA under the Trump administration. It could have been expected that Australia would take all possible measures to immunize its population as soon as possible. Observing the vaccine strategies of other high-income, high-resource countries over the same time, makes it clear that Australia could have invested its money into vaccine acquisition more wisely [92]. Moreover, investing earlier in domestic mNRA vaccine production capabilities, for instance, could have made Australia more agile in facing not only this pandemic, but also future ones. This is only made more salient by the growing evidence that this vaccine technology seems to be more resilient to mutations of the virus and is likely to become a leading technology in future vaccine development. The choice of entering into advance-purchase agreements with only three manufacturers, apart from the candidates backed by the COVAX facility, was risky. It is now clear that the immunization of the whole Australian population will be subject to the availability of mRNA vaccines and, in this regard, it appears that an advanced purchase agreement with other mRNA technology-based vaccine manufacturers, such as Moderna, would have been an advantage.

For months, Australia was able to contain the epidemic much better than most countries and might have appeared to be less in need of immediate large vaccine coverage. However, this had only been achieved at the economic and ethical cost of closing its borders. Considering the significant number of Australian nationals still stranded outside of the country, this choice is becoming problematic from a human rights perspective. This is in addition to the concerning (though clearly effective) levels of restriction of movement when a region is under a snap lockdown.

A holistic view on the relative success of the Australian economy in 2020 cannot ignore the factors which might make this recovery heterogeneous within Australian society. In particular, Indigenous people are likely to be in an industry that is most affected by the COVID-19 [102]. They are also often hired as casual workers on a temporary basis and hence, are more likely to be the first ones let go [103]. Whereas COVID-19 might have had some positive effects on work productivity by increasing the use of Information and Communication Technologies (ICT), this positive effect cannot be generalized to Indigenous populations due to the digital divide, especially in internet access [104]. The shift towards remote and online working will further exacerbate the labour inequality gap between Indigenous and non-Indigenous people.

Access to healthcare is also an important issue for the Australian indigenous people because of the higher incidence and mortality rates due to non-communicable diseases (NCDs) compared with non-Indigenous populations [105,106]. However, this access has always been harder for the Indigenous people due to a myriad of factors including discrimination, poor communication with healthcare professionals and the high cost of healthcare [107]. The long-lasting systemic discrimination faced by Indigenous Australians has been acknowledged by the Australian authorities [108]. In addition, accessing healthcare in remote areas remains a key challenge for the Australian Indigenous population [109]. Whereas the socioeconomic status of people residing in remote areas constitutes yet another barrier to accessing healthcare [110], attitudes of healthcare professionals and their perception of the difficulty of the job in working in Aboriginal health, can also affect the provision of quality services [111]. Finding and providing effective and efficient healthcare delivery in rural and remote areas of Australia is still a topic of research today [112,113,114,115], and improving access to medicines for Aboriginal and Torres Strait Islanders is also of great concern [116]. According to the Australian Institute of Health and Welfare, the Medical Benefits Schedule claim rates for tertiary services made by Indigenous Australians were 44% lower than for non-Indigenous Australians which may be due to the fact that Indigenous people are finding it significantly more difficult to find specialized care [117].

Australia’s COVID-19 response in Indigenous communities has been praised as a successful one, when compared with other countries with Indigenous populations such as Canada and the US and comparing Australia’s own experience in previous pandemics [118,119]. Inclusiveness of Indigenous people and Indigenous institutions to lead the way in pandemic planning, response and management [120] has been at the heart of the COVID-19 response. The Aboriginal and Torres Strait Islander Advisory Group on COVID-19 has been convened to provide advice on preparedness, response and recovery planning for Indigenous populations and to prevent adoption of one-size-fits-all policies [120]. The Advisory group has also developed the “Management Plan for Aboriginal and Torres Strait Islander Populations” which includes important elements of emergency response, including flexible health service delivery to ensure access to vaccination [121]. Australia’s plans to shift towards mRNA vaccines further highlights the importance of planning for vaccination accessibility in remote areas to ensure smooth vaccination of Indigenous populations, considering the special cold chain infrastructure required for mRNA vaccines.

Another issue that Australia is facing in its vaccination rollout is an increase in vaccine hesitancy towards the AstraZeneca vaccine, even amongst people over 50 years old. This was particularly so, since the controlled epidemiological situation the country experienced for months provided a sense of safety to the general population and the negative constraining effects of the pandemic on day-to-day life were relatively limited for a long time. Already before the vaccination campaign started, vaccine hesitancy was being reported in Australia [122]. It seems likely that this trend will continue. The Indigenous populations’ trust in the public health sector and the extent of their COVID-19 vaccine hesitancy is still unclear. There are examples where it has been shown that the Indigenous populations accepted to be vaccinated against HPV although not fully aware of its protective effect [123], whereas some other studies have argued that the broad mistrust in government needs to be addressed to increase participation in public health services such as colorectal cancer screening [124].

## 5. Conclusions

Overall, it is clear that in terms of dividing up resources to acquire vaccines, Australia put all its eggs in the metaphorical basket of the AstraZeneca vaccine. It is likely that, if the AstraZeneca vaccine had not been associated with any serious side effects and the ATAGI had not changed its recommendation for people below 50 years of age, the Australian vaccination rollout would have run relatively smoothly, except for the delayed start up. However, the complications and side effects from TTS associated with this vaccine cannot be ignored. There was always a risk in articulating most of the vaccination plan around one single vaccine candidate, and a more diversified strategy would have mitigated this risk. The costs would have been high but negligible compared with the economic and human cost of keeping the country’s borders closed. It is important to note that in singular situations such as the COVID-19 pandemic, where the whole world is rushing to find and create interventions to keep the global economy from collapsing and to prevent even more widespread deaths, there are many unknowns, and there are a variety of factors that can go wrong. For this reason, future pandemic preparedness plans should aim to anticipate these events and plan to cast as wide a net as possible, in terms of acquiring novel pharmaceuticals.

Another interesting point is that Australia has had access to vaccines that many other countries will not see for months, yet they have not been able to meet any of their targets. Looking at Australia’s privileged position from a different angle, one comes to this question: does it make sense, from a logistics and moral perspective, to criticize a country for not being so selfish as to make advance purchase agreements with the manufacturers of all the potentially successful vaccine candidates, when many low-income countries that are part of the COVAX facility may not get their full doses of vaccines until 2024 [125]? From this perspective, the Australian government cannot be condemned for not being more covetous of all vaccines which had received preliminary approval. From this perspective, it could be argued that the Australian vaccine strategy was fairer and more thoughtful of low- and middle-income countries. On the other hand, Australia is among the countries that secured the most doses per inhabitant in advance purchase agreements, which makes it clear that these purchasing issues were not due to financial restrictions, and the choice of not having diversified their portfolio more extensively therefore remains questionable.

Australia had extremely low daily cases for several months and its economy was able to stay afloat. The inclusion of Indigenous communities in planning and response has led to tailor-made interventions which have proven to be extremely effective. Further comparative studies between Indigenous population health outcomes in different countries after the pandemic could help highlight the extent of the success of the Australian model in that regard.

With regard to concern for anti-vaccination sentiment, Australia does not appear to have as much of a cultural precedent for these attitudes as other Western high-income countries, judging by vaccination rates [126,127]. At the time of writing, the “anti-vax” faction does not appear to make up a significant portion of the population. However, studies have also shown that vaccination hesitancy increased throughout the pandemic [128]. Tension is rising, as shown by recent protests against general anti- COVID measures, including vaccination, which attracted as many as 150 people in Melbourne [129]. It is the opinion of the authors of this paper that an unintended side effect of the strictness of Australian lockdown measures could be the further radicalization of those who are “on the fence” about the vaccine, viewing it as a further extension of the government’s attempts to control them. Overall, the nascent anti-vaccination movement in Australia is a concerning issue which needs further exploration, and could constitute an entire paper on its own, as the situation develops further.

Looking towards the future, Australia’s vaccination strategy is changing daily. The most recent changes have pivoted production towards building capacity for mRNA vaccine production, which is an advantage not only for handling the pandemic but will likely be an asset for the country’s pharmaceutical industry as a whole. However, the fruits of these efforts will not be seen until at least 2022, when in-house vaccine production will finally be able to provide mRNA vaccines. This period of time will only serve to widen the gap between Australia and its contemporaries even further. Other high-income countries have achieved high vaccination rates and many in Europe and beyond have implemented vaccine passports and opened up their borders for regular leisure travel. It is very likely that without opening up its borders, Australia may lose its “lead” on other countries, with other parts of the world moving into stronger growth positions. To make matters worse, Australia’s trade relationships are becoming increasingly unstable. Due partially to tensions over the investigations into the origins of the coronavirus, China, which was previously one of Australia’s biggest trade partners, has called to stop imports of Australian goods, and trade negotiations have halted completely [130]. This situation is deteriorating rapidly, and whereas the Australian economy is performing adequately for now, it could easily stifle growth in the future. The Australian government, therefore, needs to pay special attention to ensure that the Australian vaccination strategy is resolved, so that the country can take pride in its successes, while still taking note of the necessary lessons learned during this time, in preparation for the next pandemic.

## Figures and Tables

**Table 1:** Total COVID-19 cases and deaths in Australia, separated by each jurisdiction by the source of infection [23]. Last updated 22 May 2021.

Jurisdiction	Overseas	Locally Acquired-Contact of Confirmed Cases	Locally Acquired-Unknown Contact	Locally Acquired-Interstate Travel	Total Cases	Total Deaths
Australia	7609	17,964	4284	154	30,011	910
Australian Central Territory	95	25	1	5	124	3
New South Wales	3384	1645	451	90	5570	54
Northern Territory	167	2	0	2	171	0
Queensland	1273	264	41	23	1601	7
Southern Australia	561	152	9	26	748	4
Tasmania	85	141	5	3	234	13
Victoria	1135	15,649	3763	0	20,547	820
Western Australia	909	86	14	17	1016	9

**Table 2:** Phases of the Australian vaccine national rollout. Based on the publicly available Australian COVID-19 vaccine national roll-out strategy [58].

Phase 1a	Phase 1b	Phase 2a	Phase 2b	Phase 3
Quarantine and border workers Frontline health care workers Aged care and disability care residents and staff	>80 years 70–79 years Health care workers Aboriginal and Torres Islander people >55 Adults with underlying medical conditions and disability Critical and high-risk workers	60–69 years 50–59 years Aboriginal and Torres Islander people 18–54 Other critical and high-risk workers	Balance of adult population	<18 years if recommended
1.4 M doses	14.8 M doses	15.8 M doses	16 M doses	13.6 M doses

**Table 3:** Accessible vaccines through provisional registration [69]. Last updated 22 May 2021.

Effective Date	Sponsor	Name	Type
25 January 2021	Pfizer Australia Pty Ltd	COMIRNATY-BNT162b2 [mRNA]	mRNA
15 February 2021	AstraZeneca Pty Ltd	COVID-19 Vaccine AstraZeneca	Viral vector

**Table 4:** Comparing the States and Territories through vaccine distribution and doses administered [82]. Last updated: 17 May 2021.

	Distributed (Including Coming Weeks)	Available (Delivered Weeks 1–11, Until 9 May)	Administered (Weeks 1–12, Until 16 May)	Available Minus Administered	Estimated Dose Utilisation %
New South Wales	484,250	414,050	280,135	133,915	78%
Victoria	568,590	471,040	313,539	157,501	77%
Queensland	354,080	317,810	170,330	147,480	64%
Western Australia	213,184	187,480	130,649	56,831	80%
South Australia	137,200	116,260	80,017	36,243	79%
Tasmania	73,714	62,254	49,739	12,515	90%
Northern Territory	52,332	47,652	22,953	24,699	58%
Australian Capital Territory	60,460	53,440	38,696	14,744	82%
Jurisdiction delivered total	1,943,810	1,669,986	1,086,058	583,928	75%
Commonwealth Aged Care and Disability	340,842	304,992	296,336	8656	Fully utilised
Commonwealth Primary Care	3,256,532	2,647,632	1,717,743	929,889	75%
Total	5,541,184	4,622,610	3,100,137	1,522,473	77%
